# Hydrophilic Cross-Linked Aliphatic Hydrocarbon Diblock Copolymer as Proton Exchange Membrane for Fuel Cells

**DOI:** 10.3390/ma14071617

**Published:** 2021-03-26

**Authors:** David Julius, Jim Yang Lee, Liang Hong

**Affiliations:** Department of Chemical and Biomolecular Engineering, National University of Singapore, 10 Kent Ridge Crescent, Singapore 117585, Singapore; david_julius@hotmail.com (D.J.); cheleejy@nus.edu.sg (J.Y.L.)

**Keywords:** PEM, diblock copolymer, ATRP, hydrophilic crosslink, direct methanol fuel cell

## Abstract

This study proposes a hydrophobic and hydrophilic aliphatic diblock copolymer wherein the hydrophobic block contains glycidyl methacrylate (GMA) units that are distanced by poly(acrylonitrile) (PAN) segments to fabricate a proton exchange membrane (PEM). This diblock copolymer also known as ionomer due to the hydrophilic block comprising 3-sulfopropyl methacrylate potassium salt (SPM) block. The diblock copolymer was synthesized in the one-pot atom transfer radical polymerization (ATRP) synthesis. Subsequently, the membrane was fabricated by means of solution casting in which an organic diamine, e.g., ethylene diamine (EDA), was introduced to crosslink the diblock copolymer chains via the addition of amine to the epoxide group of GMA. As a result, the PEM attained possesses dual continuous phases, in which the hydrophobic domains are either agglomerated or bridged by the EDA-derived crosslinks, whereas the hydrophilic domains constitute the primary proton conducting channels. The in-situ crosslinking hydrophobic block by using a hydrophilic cross-linker represents the merit aspect since it leads to both improved proton conductivity and dimensional stability in alcohol fuel. To characterize the above properties, Nafion^®^ 117 and random copolymer of P(AN-*co*-GMA-*co*-SPM) were used as control samples. The PEM with the optimized composition demonstrates slightly better fuel cell performance than Nafion 117. Lastly, this diblock ionomer is nonfluorinated and hence favors lowering down both material and environmental costs.

## 1. Introduction

The development of aliphatic-based proton exchange membranes (PEMs) can benefit from rationally designed, molecularly engineered and relatively inexpensive ionomer structures. Two strategies that fall under such considerations include, firstly, well-ordered structures such as diblock [1,2], graft [3] and multiblock [4] copolymers; secondly, proton-conducting cross-linking diblock copolymer [5]. The first strategy has been extensively applied to the aliphatic-based PEMs. However, a high proton conductivity would likely have a price of dimensional stability and excessive swelling when the membranes are fully hydrated [6]. It was then reckoned that the mechanical properties of the nonfluorinated ionomer membranes could be improved by assimilating a hydrophobic cross-linkable monomer into the block copolymer chains [7]. The hydrophobic crosslinking is, however, to cause partial blockage of the hydrophilic channels to convey protons [8].

The second strategy is to make use of ionic crosslinkers such as sulfosuccinic acid (SA) so that the crosslinks of the resulting network are proton-conducting [5,9]. However, a random cross-linked membrane matrix, regardless of the ionic character of the crosslinks and the extent of crosslinking, would not provide the same connectedness as the hydrophilic channels of a well-ordered polymer structure. In addition, the ester groups, e.g., ethylene glycol dimethacrylate, in the ionic cross-links are hydrolysable by the acid moieties present in the PEM. Other than these approaches, mitigation of the proton conductivity–mechanical property trade-off in aliphatic-based PEMs is few and far between in the literature.

**Figure 1:**
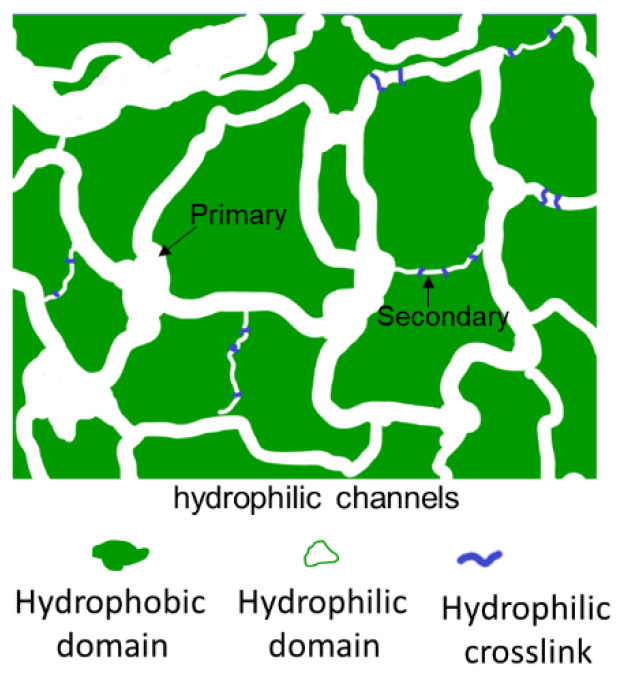
Schematic illustration showing the secondary hydrophilic channels (“waterway canals”) made possible by the hydrophilic covalent cross-linking of the diblock ionomers.

This study extends a ternary statistical copolymer of poly(acrylonitrile-*co*-glycidyl methacrylate-*co*-sulfopropyl methacrylate), P(AN-*co*-GMA-*co*-SPM) to a block copolymer chain structure, P[(AN-*co*-GMA)-*b*-SPM], which is an ionomer in functionality. The ternary ionomer chains are then tied with each other by a hydrophilic covalent crosslink that is proton conducting in nature. The design first and foremost leverages on the ordered structure of a block copolymer to synchronize the hydrophobicity–hydrophilicity difference in the membrane matrix. This is done to drive a more pronounced microscale phase separation that forms the primary hydrophilic channels and the hydrophobic domains, in which the hydrophilic covalent crosslinks are intercalated in the hydrophobic domains to constitute the secondary hydrophilic channel. This crosslinking strategy is more pertinent to strengthen the membrane matrix against swelling and high liquid fuel diffusion. A particular feature of this design is that the crosslink is capable of maintaining proton hopping as well as dimensional stability. In addition, the secondary hydrophilic channels are considered as “waterways canals” distributed in the integrated hydrophobic domains. The situation is therefore categorically different from the conventional cross-linking of the hydrophobic blocks which inevitably eliminates part of the proton transport network due to spreading local isolations, and proton conduction through the membrane has to occur over a longer path. The schematic diagram (Figure 1) illustrates the roles of the “waterways canals” in resolving the reduced proton connectivity in the hydrophilic channels. The expected outcome is therefore some relief of the conductivity–mechanical property conundrum.

The actual synthesis of a series of the SPM-based diblock ionomers was realized by a one-pot atom transfer radical polymerization (ATRP) technique conducted in a polar aprotic solvent. The selection of AN and methacrylates (SPM and GMA) aimed to the structural stability and the inherent alcohol resistance [10]. The GMA in the poly(AN-*co*-GMA) block offers the in-situ cross-linking anchors to carry out hydrophilic crosslinking. In addition to the structural reinforcement effects generally expected from cross-linking [11], the hydrophilic –OH and –NH– groups were formed from the crosslinking reaction, which could be used to transport protons and water molecules through the “waterways canals”; therefore leading to an overall improvement in proton conduction without the compensatory effect between conductivity and mechanical properties. A higher energy density output from Direct Methanol Fuel Cell (DMFC), especially at high methanol feed concentrations, was therefore made possible.

## 2. Experimental

### 2.1. Materials

3-Sulfopropyl methacrylate potassium salt (SPM, 98%), acrylonitrile (AN, ≥99%), glycidyl methacrylate (GMA, 97%), copper(I) bromide (Cu^I^Br, 98%), 2,2′-bipyridyl (bpy, 98%), 2-bromopropionitrile (BPN, 98%), ethylene diamine (EDA), ethylene carbonate (EC, 98%), dimethylsulfoxide (DMSO, HPLC-grade), diethyl ether (98%, AR-grade), methanol (99%, AR-grade), and butanol (HPLC-grade) were supplied by Sigma-Aldrich Pte Ltd., Singapore, Singapore. Sulfuric acid (95–97%, pro-analysis) was obtained from Merck, Singapore, Singapore. The inhibitors in AN and GMA monomers were removed by passing them through an alumina column from Sigma-Aldrich Pte Ltd. Cu^I^Br was purified according to the Keller–Wycoff procedure [12]. All other chemicals were used without further purification. For the comparative experiments, Nafion^®^ 112 and Nafion^®^ 117 films (Chemours, Wilmington, DE, USA) were purchased from Sigma-Aldrich Pte Ltd, St. Louis, MO, USA. The following fuel cell electrodes from BASF Fuel Cell were also used: unsupported Pt:Ru alloy (1:1) anode with 5.0 mg/cm^2^ of total metal loading and unsupported Pt cathode with 5.0 mg/cm^2^ of metal loading.

### 2.2. Synthesis of Aliphatic Diblock Ionomers by a One-Pot Atom Transfer Radical Polymerization (ATRP) Technique

**Figure 2:**
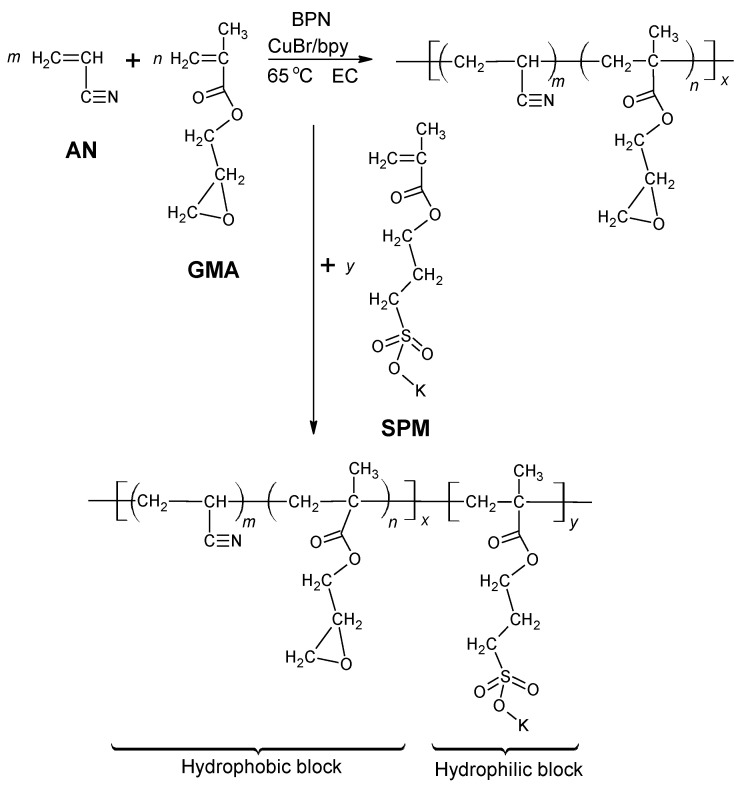
Synthesis of P[(AN-*co*-GMA)-*b*-SPM] diblock ionomer by the atom transfer radical polymerization (ATRP) technique.

The diblock ionomers were synthesized by an ATRP technique [13] with the minor modification that two solvents were used successively according to the reaction scheme in Figure 2. A mixture of 28.69 mg (0.2 mmol) of Cu^I^Br, 93.71 mg (0.6 mmol) of bpy and 15 g of EC (0.17 mol) was introduced to a 50-mL Schlenk flask. After vacuum degassing, the mixture was heated at 45 °C with stirring until it was completely molten. The solution was purged with Ar for 30 min to remove oxygen, which could interfere with the polymerization by oxidizing the radical species and the Cu(II) catalyst. The AN and GMA monomers were also separately purged and introduced to the Schlenk flask together with a specified amount of BPN initiator by a syringe. The hydrophobic block polymerization was carried out at 65 °C for 6 h.

The P[(AN-*co*-GMA)-*b*-SPM] diblock ionomers were prepared as follows: after the completion of hydrophobic block polymerization, a predetermined amount of Argon-purged SPM solution (10 mmol, 2463.2 mg in 10 mL DMSO) was added to the mixture with a syringe. Hydrophilic block polymerization was then allowed to proceed at 65 °C for 20 h. The ionomer was then separated by precipitation in an excess of a diethyl ether/methanol mixture (40/60), filtered and dried in vacuum at 40 °C for 24 h. The diblock ionomers prepared as such are identified as A_x_G_y_S-10, where x and y refer to the millimole of AN and GMA used in the starting mixture. For example, A_50_G_4_S-10 refers to the P[(AN-*co*-GMA)-*b*-SPM] ionomer synthesized from a starting mixture of 50 mmole of AN, 4 mmole of GMA, and 10 mmole of SPM. In the all compositions studied only x was varied with respect to G_4_S-10. To demonstrate the criticality of the GMA monomer, a series of P(AN-*b*-SPM) diblock ionomers were also similarly prepared and identified as A_x_S-10. For example, A_50_S-10 refers to the P(AN-*b*-SPM) ionomer synthesized from a starting mixture of 50 mmole of AN and 10 mmole of SPM.

### 2.3. Membrane Formation and Pretreatment

A specified amount of the ionomer powder was dissolved in DMSO and stirred for 4 h at room temperature. A small amount (0.5 mmole, 33.3 μL) of ethylene diamine (EDA) cross-linker was added to the solution and stirred vigorously for 5 h at room temperature to initiate cross-linking by the ring-opening reaction of the epoxide group of GMA. The solution was then decanted into a glass dish and cured at 80 °C for 48 h. A uniform transparent membrane was formed by this procedure which could easily be separated from the dish surface. The membrane was then equilibrated in 0.5 M sulfuric acid (H_2_SO_4_) for 24 h at room temperature.

### 2.4. Characterizations

#### 2.4.1. Electrochemical Analysis

The proton conductivities of the cast membranes were measured by the four-probe measurement method on an Autolab PGSTAT30 (Metrohm Autolab B.V., Utrecht, Netherlands) potentiostat / galvanostat equipped with an electrochemical impedance analyzer. The frequency range of 1 MHz to 50 Hz was used. All samples (as 1 cm × 3 cm strips) were equilibrated in deionized water for 24 h prior to the measurements. The membrane resistance was determined from the Nyquist plot of the complex impedance (Z” vs. Z’). Proton conductivity was then calculated as $\sigma =\frac{L}{RS}$, where *L* and *S* are the distance between the two electrodes (fixed at 1 cm) and the cross-sectional area of the membrane, respectively. The proton conductivity of a commercial Nafion 117 membrane measured this way was between 0.05 and 0.07 S/cm.

#### 2.4.2. Alcohol Permeability at Room Temperature

Ethanol permeability was measured at room temperature using a standard glass diffusion cell. A membrane sample was first equilibrated in deionized water for 24 h. It was then mounted between the two compartments of a glass diffusion cell. One compartment was filled with an ethanol solution of a known concentration (CA), and the other compartment (the receiving compartment) was filled with ultra-pure water (CB). The 100-μL samples were periodically drawn from the receiving compartment (every 40 min apart), and their compositions were analyzed by a Shimadzu GC-2010 (Shimadzu, Kyoto, Japan) gas chromatograph (GC) with a flame-ionization detector. The ethanol permeability was calculated under pseudo steady-state conditions by the time dependence of using the formula shown below:

(1) $$C_{B}\left(t\right)=\frac{A}{V_{B}}\frac{DK}{L}C_{A}\left(t-t_{o}\right)$$

where *A* and *L* are the membrane area and thickness respectively; *D*, *K*, and *t_o_* are the ethanol diffusivity, solubility, and the measurement time lag, respectively. The product *DK* is the membrane permeability and was calculated from the slope of the linear plot of *C_B_* against *t* according to the following equations:

(2) $$\mathrm{slope}\;=\frac{d\left(CB\right)}{dt}=\frac{A}{V_{B}}\frac{DK}{L}C_{A}$$

(3) $$\mathrm{Permeability}=DK=slope\;x\;\left(\frac{V_{B}}{A}\frac{L}{C_{A}}\right)$$

#### 2.4.3. Ion-Exchange Capacity (IEC), Alcohol and Water Uptake, and Dimensional Stability Determination

The ion-exchange capacities (IECs) of the cast membranes were determined by acid-base titrations using 1-phenolphtalein as the end-point indicator. Typically, a membrane after vacuum drying at 70 °C for 24 h was equilibrated with 1 M NaCl to fully exchange the protons (H^+^) with sodium ions. The IEC was calculated according to the formula:

(4) $$\mathrm{IEC}=\frac{M_{NaOH}x\;V_{NaOH}}{m_{dry}}$$

(i) Water uptake was calculated by the difference in weights between dry and wet membrane samples. The dry weight, *m_dry_*, was measured after a sample was placed in a vacuum oven at 80 °C for 24 h. The wet weight, *m_wet_*, was measured after equilibration of the dry sample in deionized water for another 24 h. (ii) Alcohol uptake was calculated by the difference in weights between dry and wet membrane samples. The dry weight, m_dry_, was measured after a sample was placed in a vacuum oven at 80 °C for 24 h. The wet weight, m_wet_, was measured after equilibration of the dry sample in alcohols (methanol and ethanol) for another 24 h. The dimensional stability of the membranes was estimated by the differences in the three linear dimensions (length, width, and thickness) between dry and wet samples in the liquid uptake tests.

#### 2.4.4. Examination of Membrane Morphology

The cross-section of the dry membrane was examined by FE-SEM on a JEOL JSM-6700F (JEOL, Tokyo, Japan) operating at 5 kV. All SEM samples had been vacuum-dried at 70 °C for 24 h before the examination. The cross-section was obtained by fracturing a sample membrane sample in liquid nitrogen. The SEM sample pretreatment involved Pt shadowing on a JEOL JFC-1300 (JEOL, Tokyo, Japan) auto fine coater at 10 mA for 60 s.

#### 2.4.5. MEA Preparation and DMFC Tests

The preparation of membrane electrode assemblies (MEAs) using the cross-linked diblock membranes and Nafion 117 membranes followed these procedures: for the diblock membrane, a sample was sandwiched between two 5 cm^2^ commercial electrodes and hot-pressed at 90 °C and 16 kgf/cm^2^ for 60 s. For the preparation of Nafion 117-based MEA, hot-pressing was carried out at 140 °C and 16 kgf/cm^2^ for 90 s. The resulting MEAs were kept in tightly sealed containers before use. DMFC testing was carried out in a single microcell with an effective membrane area of 25 cm^2^ supplied by Fuel Cell Technologies Inc, NM, USA. The feed to the cell was regulated by a micropump. For this study, the feed was methanol solutions in ultrapure water with the following concentrations: 1.0, 2.0, 4.0, 8.0, and 16.0 M. The methanol solution was delivered to the anode compartment at a fixed flow rate of 5 cc/min, whereas dry oxygen was fed to the cathode compartment at a fixed flow rate of 50 cc/min. The DMFC performance was evaluated at two temperatures: 30 and 50 °C. As part of the cell conditioning, the cell was rested in the open circuit condition for 30 min before measurements were taken.

## 3. Results and Discussion

### 3.1. Characteristics of the Diblock Ionomer Membranes

It has been hypothesized that high proton conductivity and good mechanical properties of many of the state-of-the art PEMs are consequential upon an ordered polymer structure [14]. In Nafion, for example, the perfluorosulfonate side chains are grafted onto the poly(tetrafluoro ethylene)-main chains at nearly regular intervals. This unique chain structure, with large hydrophobicity-hydrophilicity difference between different parts of the polymer, drives phase separation at nanoscale upon hydration, forming extensively connected proton-conducting channels throughout the membrane matrix [15]. Similarly, alternative aliphatic PEMs prepared from an ordered polymer structure, such as the diblock, multiblock or branch/graft structure, also showed good PEM properties as a result of hydrophobicity–hydrophilicity-driven phase separation [16]. The extensiveness of the connectivity of the hydrophobic and hydrophilic domains is determined by the extent and the length scale of phase separation which should be dependent on the hydrophobicity–hydrophilicity difference in the ionomer.

There are two hurdles in replicating a Nafion-like architecture in nonfluorinated hydrocarbon polymers such as aliphatic diblock ionomers with alternating hydrophobic and hydrophilic blocks. Firstly, the perfluorocarbon chains of Nafion are far more hydrophobic than any other hydrocarbon chains. Hence the hydrophilicity–hydrophobicity driven phase separation in hydrocarbon-based ionomers would not be as extensive and would not occur at the same small length scale as that in Nafion. The proton conducting channels in the former are therefore more tortuous and there is more likelihood for partial blockages [17]. Secondly, the hydrophilic segment of Nafion is much shorter than its perfluoro block and the overall molecular weight of Nafion is also far smaller than what hydrocarbon ionomers usually should have, so that Nafion molecules could readily undergo self-assembling to generate highly divided double continuous phases.

**Figure 3:**
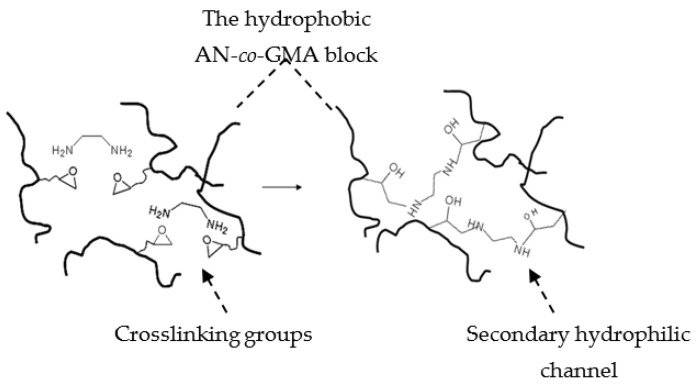
Schematic illustration of the cross-linking between the hydrophobic poly(acrylonitrile) (PAN) blocks and the formation of the secondary hydrophilic channels in the merged hydrophobic domains.

To circumvent the hurdles, the P(AN-*co*-GMA)-*b*-SPM diblock ionomer (Figure 2), synthesized by an ATRP technique in an EC/DMSO dual-solvent system, lays out the bicontinuous hydrophilic and hydrophobic domains due to the orderliness in a block structure with alternating hydrophobic and hydrophilic properties. In-situ crosslinking was introduced during membrane casting to form a covalently cross-linked network with hydrophilic character (Figure 3). A designated cross-linker such as an organic diamine [R(NH_2_)_2_], EDA, was added to the casting solution. Upon drying the cast membrane, the reaction between the diamine molecules and the pendant epoxide groups of GMA established a covalently bonded network between the hydrophobic (poly(acrylonitrile) (PAN)-*co*-GMA) blocks, allowing freestanding membranes to be made.

Acronym: A_x_G_y_S–10; Acrylontrile Glycidyl methacrylate Sulfopropyl methacrylate, where x and y are millimole numbers with respect to 10 mmole S.

The in-situ crosslinking is a key feature in this PEM design. The cross-linked network helps to integrate neighboring hydrophobic blocks into a continuum, thus strengthening the membrane matrix. By comparison, the ionomers prepared from diblock P(AN-*b*-SPM) without GMA failed to form free-standing membranes (Table 1). The reaction between the epoxide group of GMA and diamine formed not only the covalent aminoethanolic linkages but also hydrophilic –OH and –NH– groups that imparted a hydrophilic character to the crosslinks. The association of the hydrophilic crosslinks formed secondary hydrophilic channels (“the waterways canals”) in the integrated hydrophobic domains to support the transport of protons and water molecules across the hydrophobic domains (Figure 3).

It is generally known that cross-linked PEMs improve the membrane dimensional stability and swelling resistance because of a more compact membrane structure [11] which, however, increases the resistance to proton conduction. Proton conductivity is generally decreased by the increase in hydrophobicity caused by covalent cross-linking which isolated some of the hydrophilic domains [18]. This issue could be addressed by hydrophilic covalent crosslinking which does not block the diffusion of water molecules and protons. It is hypothesized that the introduction of hydrophilic character in the covalent crosslinks led to the creation of secondary hydrophilic channels in the integrated hydrophobic domains which were connected to the primary hydrophilic channels. They functioned as bypasses to shorten the proton transport paths between hydrophobic regions.

Table 2 shows the comparison of PEM properties between cross-linked diblock ionomer PEMs and a Nafion 117 membrane. The high proton conductivity of the cross-linked diblock ionomer membranes can clearly be attributed to the ordered ionomer structure and hydrophilic covalent cross-linking. Both factors are considered equally important in supporting proton transport through the membranes. A previous study has shown how the blocks in ordered aliphatic ionomers may be designed to provide high proton conductivity through the creation of a continuous ionic cluster network [6]. However, the membranes made from such aliphatic block ionomers also swelled excessively in water. The gain in the freedom for segmental motion of the hydrophilic blocks also increased the mobility of the hydrophobic blocks; causing more frequent transitory chain entanglements across the domain boundaries. The resulting increase in the constriction and tortuosity of the hydrophilic channels explained the low IEC value measured experimentally. Furthermore, the highly swollen membranes, with their rather weak mechanical proprieties, could not form MEAs. Hence the design of ordered ionomer structures should not be optimized only for a particular property but rather a cache of properties with additional considerations such as effective IEC and MEA manufacturability.

**Figure 4:**
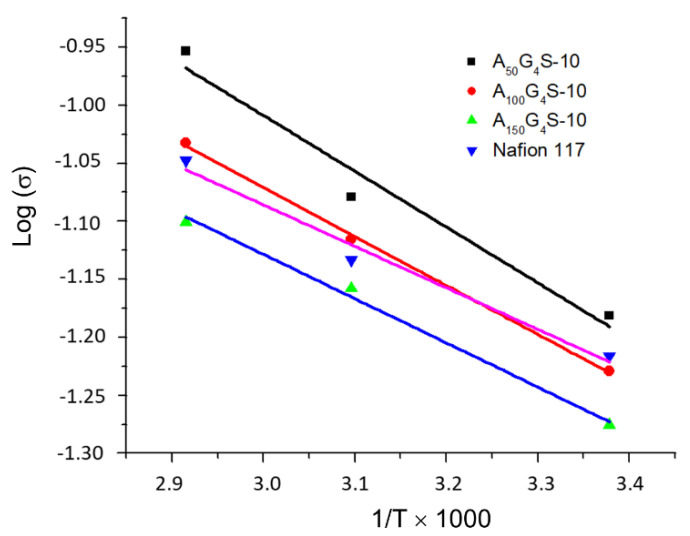
Arrhenius plot of proton conductivities of fully hydrated cross-linked diblock ionomer membranes and Nafion 117.

Figure 4 shows the Arrhenius plot of proton conductivities of the P(AN-*co*-GMA)-*b*-SPM membranes and a Nafion 117 membrane as a function of temperature. The proton conductivities of the cross-linked diblock membranes were comparable to that of the Nafion 117 membrane over the temperature range indicated and increased with temperature. In some cases, a proton conductivity higher than Nafion was also possible. Figure 4 also highlights the relationship between proton conductivity and the composition of the diblock ionomers. It is apparent that proton conductivity decreases with the increase in the hydrophobic/hydrophilic ratio of the diblock ionomer. Hence, the ionomer with the lowest hydrophobic/hydrophilic ratio, i.e., the A_50_G_4_S-10, forms a membrane with the highest proton conductivity. The dependence of membrane proton conductivity on IEC and water uptake is a well-documented observation. A membrane with high IEC would be highly hydrophilic, and consequently absorbs the most water. This is to be expected since the affinity for water is increased by a larger number of ionizable sulfonic acid groups in the ionomer. The A_50_G_4_S-10 membrane, which shows the highest amount of water uptake in Table 2, followed exactly this trend as it also has the highest IEC. This can be attributed to a greater extent of phase separation in membranes made from ionomers with low hydrophobic/hydrophilic ratios. With an increasing extent of phase separation, a more connected and continuous network of hydrophilic channels could be formed to support the transport of protons. On the other end of the spectrum, for the membrane prepared from the largest hydrophobic/hydrophilic ratio, A_150_G_4_S-10, the connectivity of primary hydrophilic channels network is probably impeded by the long hydrophobic chains, which decreased the IEC and hence the associated hydrophilicity; thereby increasing the resistance to proton conduction. In the meantime, A_150_G_4_S-10 presents the minimum methanol diffusivity of the three specimens studied.

The activation energies calculated from the Arrhenius plots of proton conductivity (Figure 4) are summarized in Table 3. The values for the cross-linked diblock ionomer membranes are all in the range of 0.076–0.095 eV; similar to the activation energy of Nafion 117 (0.085 eV measured). Activation energy is commonly used to deduce the dominant mechanism for proton transport. For Nafion membranes the Grotthus mechanism is dominant if the activation energy falls within the range of 0.09–0.012 eV) [19,20]. Hence, the activation energy similar to that of proton transport in Nafion may be used to infer the predominance of the Grotthus mechanism in the diblock ionomer membranes. This could in turn be related to the structure and distribution of the proton transport channels, such as the domain-boundary structure as shown below by the electron micrographs.

### 3.2. Examination of Liquid Uptake and Dimensional Stability

Dimensional stability against liquid swelling is an important factor to consider in the fabrication of MEAs. Excessive membrane swelling due to a high liquid uptake can cause significant changes in the membrane dimensions that lead to the deconstruction of the MEA structure. A classic failure mode is the delamination of the catalyst layers from the membrane layer resulting in poor or no fuel cell performance. Delamination indicates poor interfacial adhesion between the electrodes and the PEM in a MEA. This problem is common in hydrocarbon-based PEMs where material properties can be significantly different from those of Nafion which is used as a binder in the electrodes.

**Figure 5:**
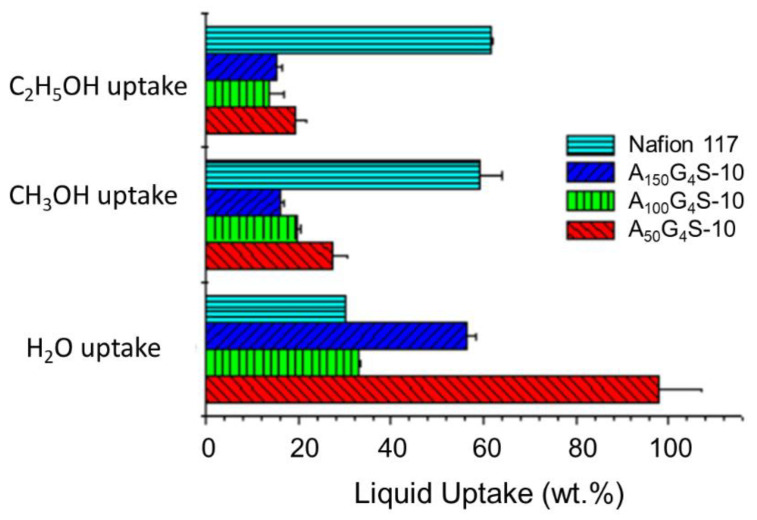
Uptakes of different liquids by the cross-linked diblock ionomer membranes. A Nafion 117 membrane is included for comparison.

Figure 5 shows the liquid uptake of the cross-linked P(AN-*co*-GMA)-*b*-SPM diblock ionomer membranes in comparison with the Nafion 117 membrane. Relative to the latter, the cross-linked diblock ionomer membranes had higher water uptake but lower alcohol (methanol and ethanol) uptakes. The higher water uptake could be attributed to the strong hydrophilicity of the sulfonic acid group in SPM and hydrophilicity of the –OH and –NH– groups in the covalent cross-links. Among the diblock membranes, water uptake increased with decreasing hydrophobic/hydrophilic ratio. This is indication of the changes in water affinity which resulted in more severe membrane swelling.

In addition, the liquid uptake was also dependent on the type of alcohol solutions. Specifically, methanol uptake was much lower than ethanol uptake. This is because relative to methanol, ethanol has a solubility parameter (δ) which is closer to the solubility parameters of most hydrocarbon monomer units (e.g., styrenic, acrylic, halide, and aromatic), and hence it can better solvate the hydrophobic segments. In addition, the reduced swelling of the cross-linked diblock membranes in alcohol solutions was expected, considering the poor solubility of acrylic polymers such as PAN (solubility parameter, δ = 12.7) and MMA (δ = 9.5) in both methanol (δ = 7.4) and ethanol (δ = 9.5) [21]. Indeed, the extremely low swelling in both methanol and ethanol solutions is similar to that of PAN-based proton exchange membranes [9,22]. Such improved swelling characteristics are one of the innate advantages of acrylic classes of polymers for PEM design.

**Figure 6:**
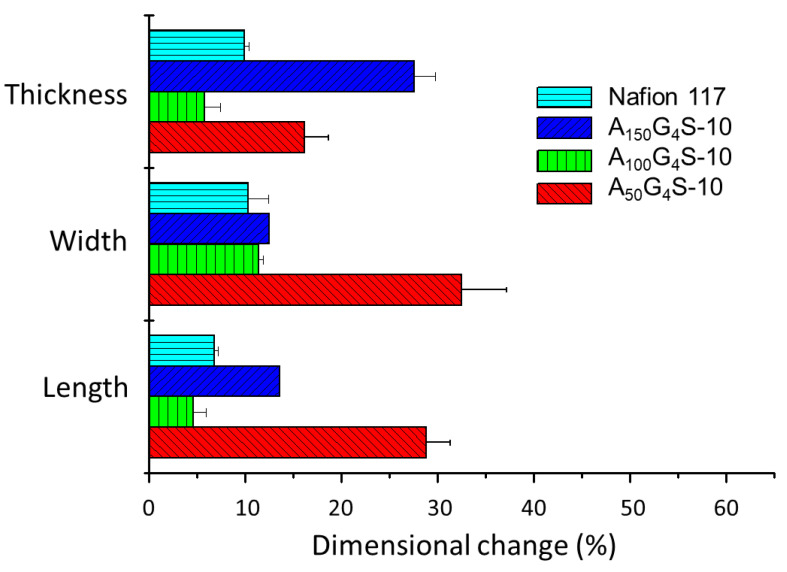
Dimensional stability of the cross-linked diblock ionomer membranes in water. A Nafion 117 membrane is included for comparison.

**Figure 7:**
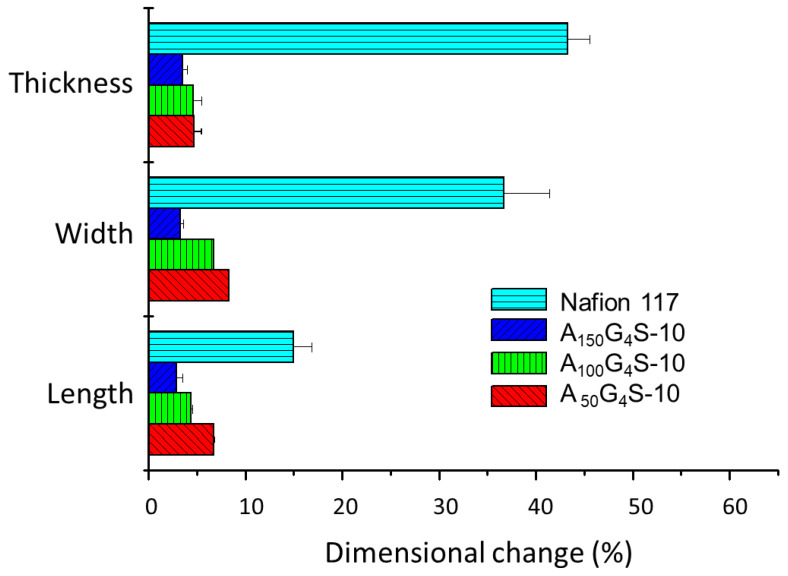
Dimensional stability of the cross-linked diblock ionomer membranes in pure methanol (29.7 M). A Nafion 117 membrane is included for comparison.

**Figure 8:**
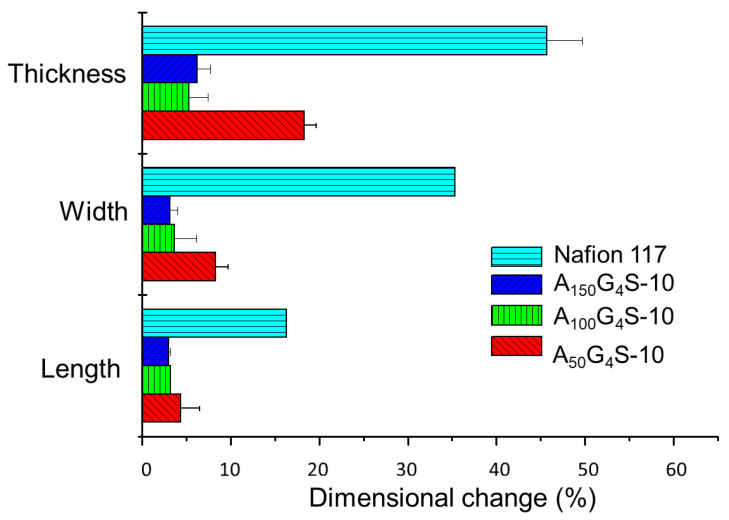
Dimensional stability of the cross-linked diblock ionomer membranes in pure ethanol (20.6 M). A Nafion 117 membrane is included for comparison.

Figure 6, Figure 7 and Figure 8 show the swelling of the cross-linked diblock ionomer membranes and a Nafion 117 membrane in the in-plane (length and width) and through-plane (thickness) directions in water and pure alcohols (methanol and ethanol). The A_50_G_4_S-10 membrane shows the highest liquid uptake (Figure 5) and swelling among the three specimens, and is also greater in comparison with the other two diblock membranes. It is postulated that A_50_G_4_S-10, with the shortest hydrophobic block in an ordered polymer structure, induces the formation of wider hydrophilic channels in the membrane and a high connectivity of the hydrophilic domains. Consequently, a larger amount of water could be accommodated.

**Figure 9:**
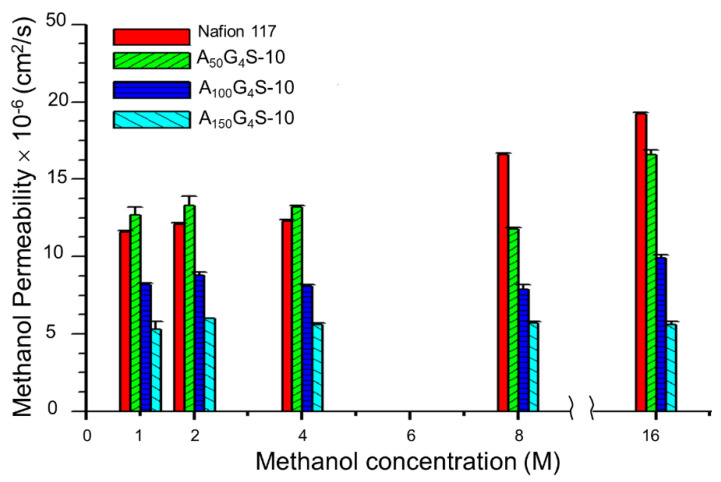
Effect of methanol concentration on the methanol permeability of cross-linked diblock ionomer membranes. A Nafion 117 membrane is included for comparison.

**Figure 10:**
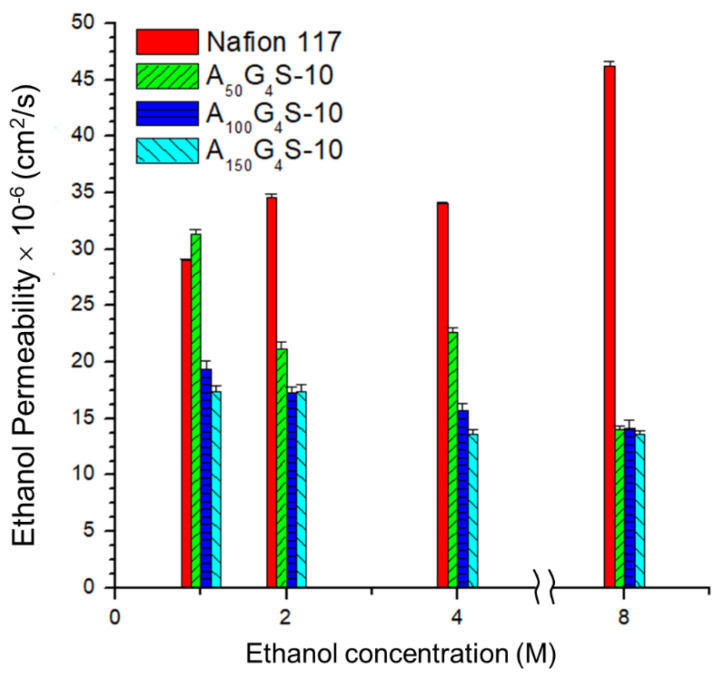
Effect of ethanol concentration on the ethanol permeability of cross-linked diblock ionomer membranes. A Nafion 117 membrane is included for comparison.

Figure 9 and Figure 10 show, respectively, the methanol and ethanol permeabilities of the cross-linked diblock ionomer membranes and a Nafion 117 as functions of alcohol concentration. The measured methanol and ethanol permeabilities of Nafion117 in 1M alcohols are 1.17 × 10^−6^ and 2.9 × 10^−6^ cm^2^⋅s^−1^, respectively, and are in good agreement with the literature values. Except for the A_50_G_4_S-10 membrane, the cross-linked diblock membranes generally showed much greater alcohol resistance than the Nafion 117 membrane. The relatively poor showing of the A_50_G_4_S-10 membrane could be attributed to excessive swelling caused by a higher proportion of sulfonic acid groups in this ionomer. In these Figures, alcohol permeability of Nafion 117 presents an obvious increasing trend compared to the other membranes in question with the increase in alcohol concentration.

This trend may be attributed to the ease of swelling of Nafion in alcohols, thereby allowing alcohol molecules to be able to transport through hydrophilic domains in the membrane. However, the extent of swelling is lower in the cross-linked diblock ionomer membranes. Furthermore, the swelling characteristics in methanol and ethanol are completely different. While methanol concentration does not apparently affect methanol permeability, ethanol permeability is significantly lowered in higher ethanol concentrations. These opposite trends could be understood in terms of solvent solvation effects. The hydrophobic (AN-*co*-GMA) block can be partially solvated by ethanol while the hydrophilic SPM block is completely solvated by water. Increase in ethanol concentration could therefore promote the segmental motion of the hydrophobic blocks through the ethanol solvation effect. On the other hand, the decrease in the water content at the same time would reduce hydration of the hydrophilic domains and hence the segmental motion of the hydrophilic blocks. It is noteworthy to point out that the decreased mobility of the hydrophilic blocks could also impede the segmental motion of the hydrophobic blocks; contravening the increase in hydrophobic mobility due to the ethanol solvation effect. For example, at high ethanol concentrations (8 M) the reduced water content in the hydrophilic domains constrains indirectly the segmental motion of the hydrophobic blocks, and inhibited the transport of ethanol molecules through the hydrophobic domains of the diblock ionomer as a result. However, at low ethanol concentrations (1 M), the water content in the solution is sufficient to cause excessive membrane swelling due to the hydrophilic-block-induced segmental movement of the hydrophobic blocks.

Although the presence of water in PEM is critically important for proton transport, excess water can cause undue swelling and increase alcohol permeability. Hence swelling is more severe with the higher the hydrophilicity of the ionomer (at a high sulfonic acid content). In general, alcohol permeability and water uptake of ionomer membranes are positively correlated. Nafion membranes, for instance, have been known to have high alcohol permeability and significant water uptake. High alcohol permeability could, in principle, be suppressed by designing an ionomer structure that minimizes water uptake and enhances the cohesive forces in the hydrophobic domains to oppose swelling. Nevertheless, for the P(AN-*co*-GMA)-*b*-SPM diblock ionomer membranes, an alcohol permeability lower than that of Nafion was still possible at water uptakes much higher than Nafion (Table 2). These observations suggest that the diblock ionomer membranes have stronger intrinsic alcohol resistance, not only due to the use of alcohol-resistant constituents (PAN and acrylates) and hydrophilic covalent cross-links, but also a domain structure through the diblock design). The domain structure imparted more regularity in the membrane structure through the formation of connected hydrophilic channels. Each domain consists of several A_x_G_y_ blocks, in which strong association of the AN segments and covalent cross-linking bonds between the GMA units provided mechanical stability as well as low alcohol permeability. It has been demonstrated that a membrane which is cross-linked to constrain swelling can effectively also reduce alcohol permeation [11,23]. Such was the benefit of hydrophilic covalent cross-linking in the current ionomer design.

### 3.3. Microstructures of the Dual Phase Membrane

**Figure 11:**
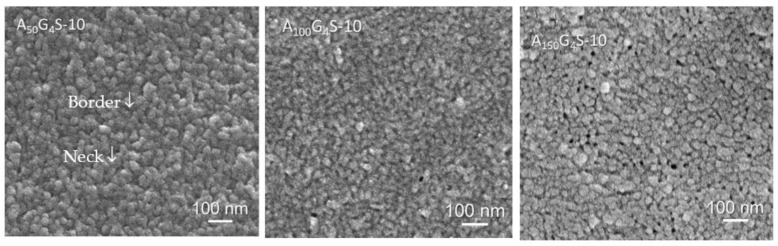
FE-SEM images of the three A_x_G_y_S-10 cross-linked diblock membranes.

**Figure 12:**
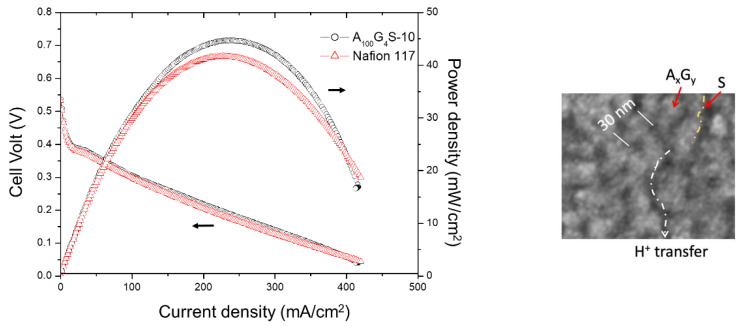
Performance of the A_100_G_4_S-10 cross-linked diblock ionomer membrane electrode assembly (MEA) and a Nafion 117 MEA tested in a DMFC running at 30 °C with a 4.0 M methanol feed (flow rate: 5 cc/min for MeOH and 50 cc/min for dry oxygen). Inset: showing the influence of microstructure on the cell performance.

Electron microscopy was used to examine the microstructures of membrane, and from which to infer some the property changes in ionomers with different hydrophobic/hydrophilic ratios. From Table 2 one can conclude that room temperature proton conductivity and water uptake decreased with the increase in hydrophobic/hydrophilic ratio. A longer hydrophobic block, such as that in A_150_G_4_S-10, is expected to form larger and more rigid hydrophobic domains and more likely to cause discontinuities in the hydrophilic domains. On the contrary, the A_50_G_4_S-10 membrane with a shorter hydrophobic block would form less rigid hydrophobic domains and indirectly improved the connectivity and continuity of the hydrophilic domains. The hypothesis that the hydrophobic/hydrophilic ratio affected the formation of different ionic aggregates was tested by examining the cross-sections of the diblock membrane with FE-SEM (Figure 11). The images show the nano-domains (≈30–50 nm) morphology in all the membrane specimens. It is also worthy of note that A_100_G_4_S-10 presents the finest phase separation morphology, meaning that GMA units are most uniformly distributed in the hydrophobic A_100_G_4_ block. Thus, the crosslinking could most effectively limit the aggregation of AN segments, and hence the highest boundary region where the hydrophilic S block locates as illustrated (yellow) in the inset of Figure 12. There are both boundaries and necks between domains [24]. The boundary phase is regarded as primary hydrophilic channels whereas the necks as the secondary hydrophilic channel and the hydrophobic coalescing joints. In addition, with the increase in the dose of the hydrophobic block to x = 100, the boundary phase characteristic fades away and the domain sizes reduce, implying more hydrophilic coalescence occurs. Finally, when x = 150, the membrane reveals pits where the loci are formed of the aggregated hydrophilic blocks because the dilution of hydrophilic crosslinks. Additional FE-SEM images of cross-linked diblock copolymer membranes (in nano-scale) have been included in the Appendix A.

### 3.4. Assessment of DMFC Performance

The mechanical properties of PEM are often evaluated in terms of their ability to form freestanding membranes and to withstand the tensile measurements under certain test conditions. However, such ability does not directly translate into practical utility of the PEMs for fuel cell applications. It is current opinion that an important requirement for PEMs is their viability to be fabricated into a membrane electrode assembly (MEA), and the performance of that MEA in real fuel cell applications. The diblock ionomer membranes developed in this study were therefore fabricated as the MEAs for DMFCs. While all of the ionomers could form freestanding membranes, the A_50_G_4_S-10 membrane was unable to form a MEA because of its high swelling characteristics and poor dimensional stability. The high-water uptake in the hydrated A_50_G_4_S-10 membrane sample was unable to maintain any dimensional stability during MEA fabrication. The loss of water due to hot-pressing resulted in a very brittle and easily fragmented membrane.

The performance of the MEA of cross-linked diblock membrane represented by the A_100_G_4_S-10 membrane was evaluated in a single-stack DMFC and benchmarked against the performance of a commercial Nafion 117-based MEA under identical test conditions: an operating temperature of 30 °C and a feed of 4 M aqueous methanol solution to the anode (Figure 12).

**Figure 13:**
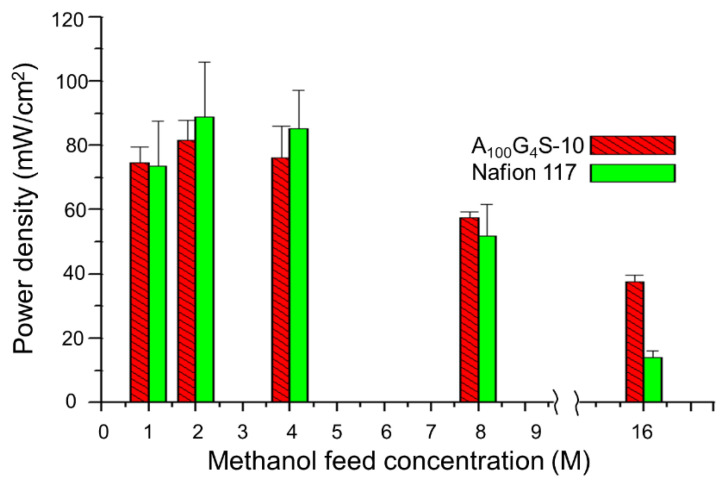
Maximum power density with different methanol feed concentrations at 50 °C. All tests were carried out after conditioning the cell for 30 min at the open-circuit condition (flow rate: 5 cc/min for MeOH and 50 cc/min for dry oxygen).

It was found that both MEAs exhibited rather similar activation losses and ohmic resistance in the overall cell reaction. However, the A_100_G_4_S-10-based MEA could deliver a slightly higher maximum power density. The improvement could be attributed to the better methanol blocking property of the A_100_G_4_S-10 membrane. The higher performance of the A_100_G_4_S-10-based MEA also prevailed at a higher operating temperature of 50 °C using feeds of different methanol concentrations at the anode and dry oxygen at the cathode as reported in Figure 13. The difference between the power outputs of the A_100_G_4_S-10 and Nafion 117 membranes was small except at very high methanol concentrations (16M). This trend is most apparent in Figure 13, which plots the maximum power density against the methanol concentration. Clearly, the Nafion 117 membrane was unable to sustain the same fuel cell performance at high methanol concentrations in the feed, whereas the A_100_G_4_S-10 membrane had no such failing. It should also be mentioned that the thickness of the Nafion 117 membrane was about double that of the diblock membrane. On the other hand, a separate experiment using MEA made from a thinner Nafion membrane (Nafion 112, with thickness of 50 μm, which is about one quarter of the thickness of the Nafion 117 membrane) had experienced an even greater loss of power density (maximum power density of Nafion 112 was 12 mW/cm^2^). For comparison, the maximum power densities of A_100_G_4_S-10 and Nafion 117 membranes were 41 and 16 mW/cm^2^, respectively, while the open circuit voltage (OCV) of A_100_G_4_S-10 and Nafion 117 membranes was found to be about the same at 0.54 V. Hence, the power density loss should correlate with the methanol blocking ability of the membrane, and not to the ohmic resistance of the membrane. The better performance of A_100_G_4_S-10 is reckoned to have the root cause in its finest hydrophobic grains (A_x_G_y_) and highest hydrophilic boundary (S) illustrated in the inset of Figure 12). Such morphology would make methanol swelling difficult just because of the highest spatial packing of the hydrophobic grains.

## 4. Conclusions

We report an alternative design to achieve a nonfluorinated PEM by solution casting technique, in which the in-situ crosslinking of the hydrophobic blocks of a diblock copolymer, poly[(AN-*co*-GMA)-*b*-SPA], is arranged by solution casting and thermal curing. This results in the secondary “waterways channels” in the hydrophobic domains with the exception of the primary one formed of the hydrophilic SPA blocks. The resulting freestanding PEM delivers improved proton conductivity and alcohol resistance (with respect to Nafion 117) in addition to promising mechanical properties. The hydrophobic AN-*co*-GMA to the hydrophilic SPA ratio was an important structural parameter as it determines both proton conductivity and fuel permeability of the membrane. An optimal hydrophobic/hydrophilic ratio in the ionomer leads to a balance amidst all the requisite PEM properties. The swelling and dimensional stability of the cross-linked diblock ionomer membranes depend not only on polymer composition and hence the ionomer structure, but also the type of alcohol solutions (methanol or ethanol).

## Figures and Tables

**Table 1:** Compositions and film-forming properties of A_x_S-10 and A_x_G_y_S-10 diblock ionomer systems.

Copolymer	[AN] (mmol)	Film Formation without In-Situ Cross-Linking	Film Formation after In-Situ Cross-Linking
A_50_S-10	50	Failed	Failed
A_100_S-10	100	Failed	Failed
A_150_S-10	150	Failed	Failed
A_50_G_4_S-10	50	Failed	Success
A_100_G_4_S-10	100	Failed	Success
A_150_G_4_S-10	150	Failed	Success

**Table 2:** Comparison of proton exchange membrane (PEM) properties between the cross-linked diblock ionomer membranes and Nafion 117 membrane.

Membrane	Thickness ^a^ (μm)	Proton Conductivity ^b^	Methanol Permeability ^c^	Characteristic Factor ^d^	Water Uptake	IEC
		(mS cm^−1^)	×10^−7^ cm^2^/s	×10^4^ (S cm/s)	(%)	(mmol/g)
A_50_G_4_S-10	96	66 ± 2.7	12.7	5.0	97.9	1.15
A_100_G_4_S-10	105	62 ± 3.0	8.2	7.6	32.8	0.87
A_150_G_4_S-10	86	53 ± 2.5	5.3	10.0	56.2	0.70
Nafion 117	200	62 ± 3.0	11.6	5.3	30.0	0.90

^a^. After acidification treatment (hydrated); ^b^. Test conditions: 23 °C and 70% RH; ^c^. [MeOH] = 1 M; ^d^. Characteristic factor = (Proton conductivity)/(Methanol permeability).

**Table 3:** Activation energies of cross-linked diblock ionomer membranes. A Nafion 117 membrane is included for comparison.

Membrane	Activation Energy	
	(J/mole)	(eV)
A_50_G_4_S-10	9203.6	0.095
A_100_G_4_S-10	8688.1	0.090
A_150_G_4_S-10	7299.7	0.076
Nafion 117	8181.8	0.085

**Figure A1:**
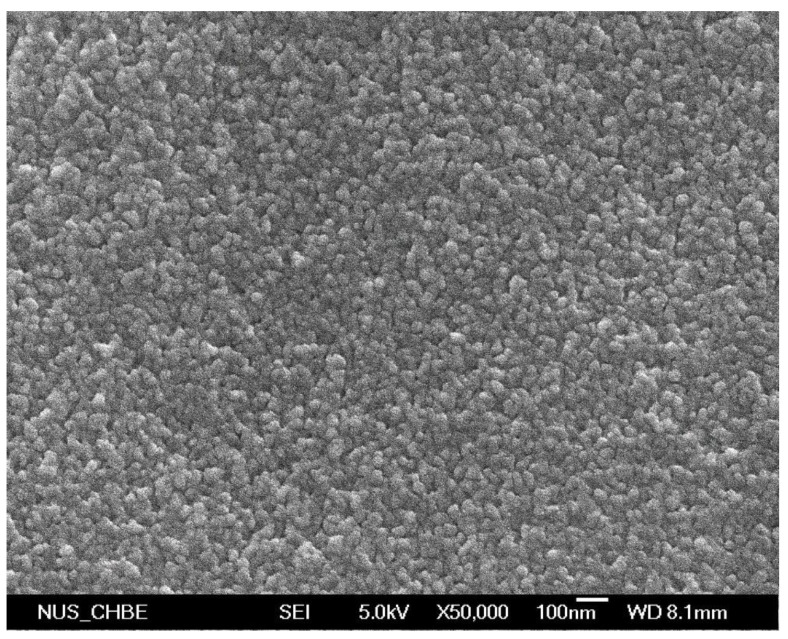
FE-SEM images of cross-linked diblock A50G4S-10 membrane (magnification 50,000×).

**Figure A2:**
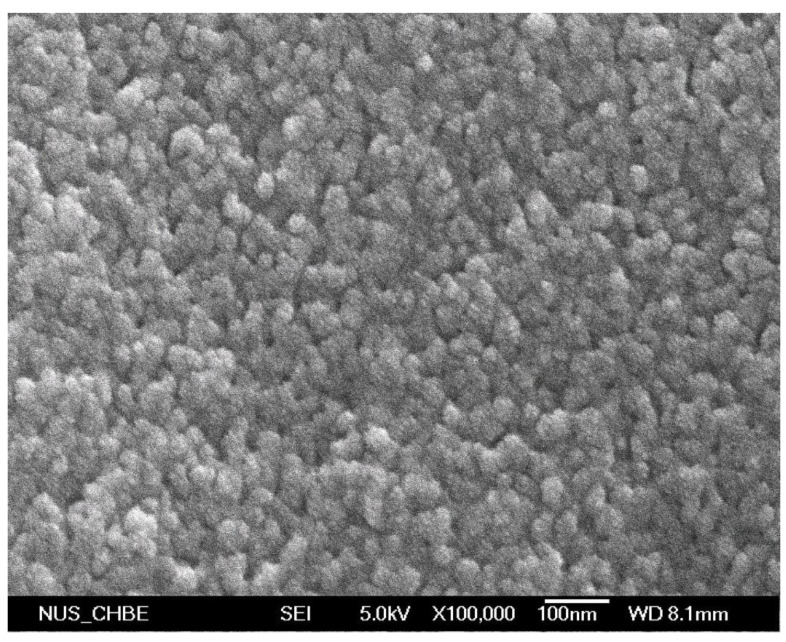
FE-SEM images of cross-linked diblock A50G4S-10 membrane (magnification 100,000×).

**Figure A3:**
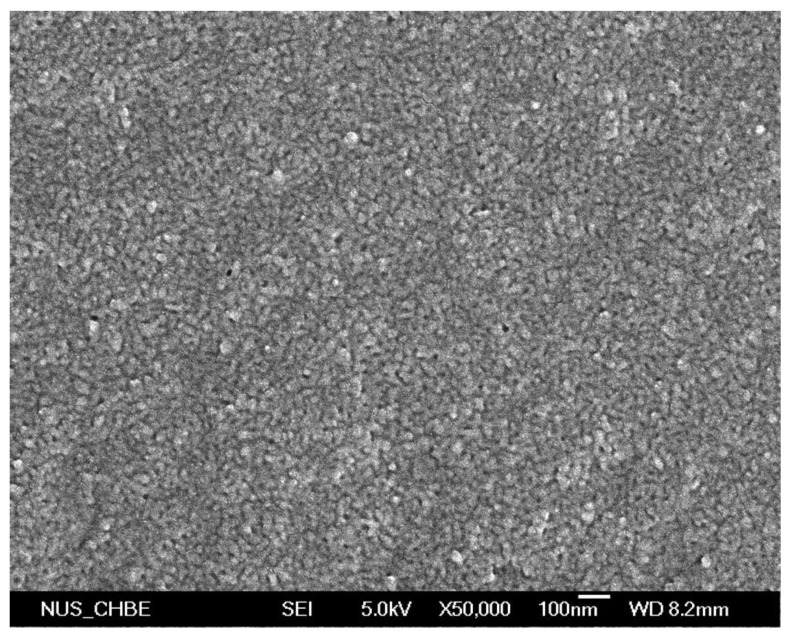
FE-SEM images of cross-linked diblock A100G4S-10 membrane (magnification 50,000×).

**Figure A4:**
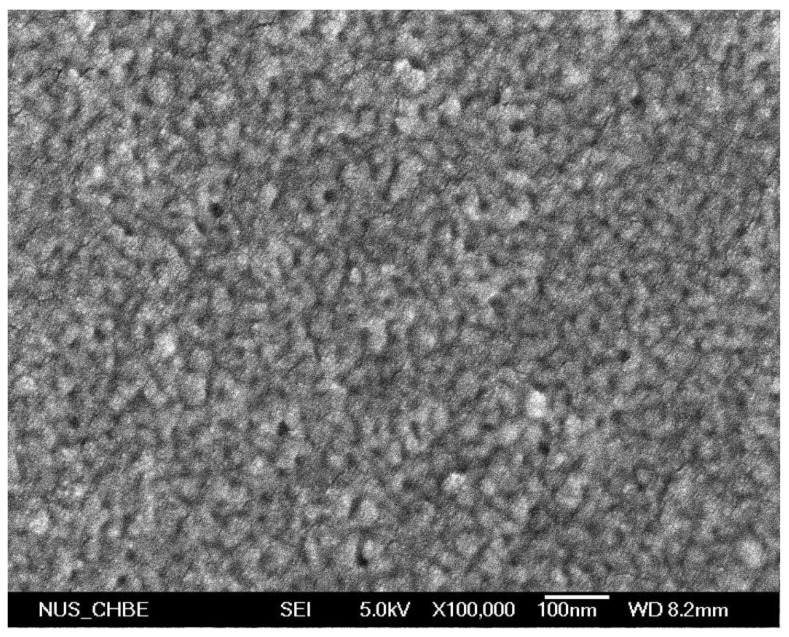
FE-SEM images of cross-linked diblock A100G4S-10 membrane (magnification 100,000×).

**Figure A5:**
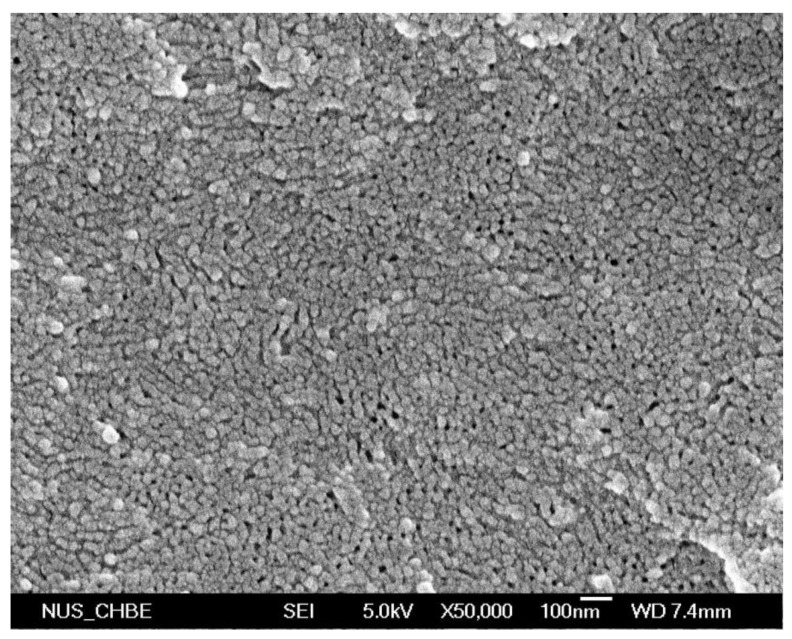
FE-SEM images of cross-linked diblock A150G4S-10 membrane (magnification 50,000×).

**Figure A6:**
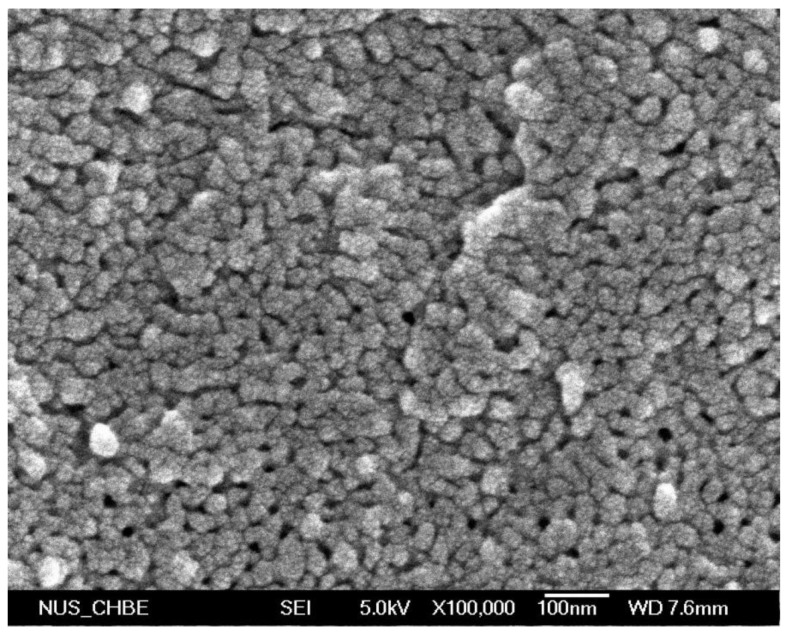
FE-SEM images of cross-linked diblock A150G4S-10 membrane (magnification 100,000×).

## Data Availability

Excluded.

## Appendix A

SEM-images to show the micro-morphologies of the membranes prepared.

## References

[1] Erdogan T., Unveren E.E., Inan T.Y., Birkan B. (2009). Well-defined block copolymer ionomers and their blend membranes for proton exchange membrane fuel cell. J. Membr. Sci..

[2] Lee W., Kim H., Lee H. (2008). Proton exchange membrane using partially sulfonated polystyrene-b-poly(dimethylsiloxane) for direct methanol fuel cell. J. Membr. Sci..

[3] Ding J., Chuy C., Holdcroft S. (2002). Enhanced Conductivity in Morphologically Controlled Proton Exchange Membranes: Synthesis of Macromonomers by SFRP and Their Incorporation into Graft Polymers. Macromolecules.

[4] Elabd Y.A., Walker C.W., Beyer F.L. (2004). Triblock copolymer ionomer membranes: Part II. Structure characterization and its effects on transport properties and direct methanol fuel cell performance. J. Membr. Sci..

[5] Lee D.K., Kim Y.W., Choi J.K., Min B.R., Kim J.H. (2008). Preparation and characterization of proton-conducting crosslinked diblock copolymer membranes. J. Appl. Polym. Sci..

[6] Tsang E.M.W., Zhang Z., Shi Z., Soboleva T., Holdcroft S. (2007). Considerations of Macromolecular Structure in the Design of Proton Conducting Polymer Membranes: Graft versus Diblock Polyelectrolytes. J. Am. Chem. Soc..

[7] Fisher A.M. (2003). Polymer Electrolyte Membrane and Method of Fabrication. U.S. Patent.

[8] Hofmann M.A., Ambler C.M., Maher A.E., Chalkova E., Zhou X.Y., Lvov S.N., Allcock H.R. (2002). Synthesis of Polyphosphazenes with Sulfonimide Side Groups. Macromolecules.

[9] Rhim J.-W., Park H.B., Lee C.-S., Jun J.-H., Kim D.S., Lee Y.M. (2004). Crosslinked poly(vinyl alcohol) membranes containing sulfonic acid group: Proton and methanol transport through membranes. J. Membr. Sci..

[10] Chiang W.-Y., Lin Y.-H. (2003). Properties of modified polyacrylonitrile membranes prepared by copolymerization with hydrophilic monomers for water-ethanol mixture separation. J. Appl. Polym. Sci..

[11] Fu R.Q., Hong L., Lee J.Y. (2008). Membrane Design for Direct Ethanol Fuel Cells: A Hybrid Proton-Conducting Interpenetrating Polymer Network. Fuel Cell.

[12] Keller R.N., Wycoff H.D., Fernelius W.C. (1946). Copper(I) Chloride. Inorganic Syntheses.

[13] Matyjaszewski K., Jo S.M., Paik H.J., Gaynor S.G. (1997). Synthesis of well-defined polyacrylonitrile by atom transfer radical polymerization. Macromolecules.

[14] Yang Y., Holdcroft S. (2005). Synthetic Strategies for Controlling the Morphology of Proton Conducting Polymer Membranes. Fuel Cell.

[15] Mauritz K.A., Moore R.B. (2004). State of Understanding of Nafion. Chem. Rev..

[16] Ding J., Chuy C., Holdcroft S. (2002). Solid Polymer Electrolytes Based on Ionic Graft Polymers: Effect of Graft Chain Length on Nano-Structured, Ionic Networks. Adv. Funct. Mater..

[17] Elabd Y.A., Napadensky E., Walker C.W., Winey K.I. (2006). Transport Properties of Sulfonated Poly(styrene-*b*-isobutylene-*b*-styrene) Triblock Copolymers at High Ion-Exchange Capacities. Macromolecules.

[18] Kerres J.A. (2005). Blended and Cross-Linked Ionomer Membranes for Application in Membrane Fuel Cells. Fuel Cell.

[19] Pei H., Hong L., Lee J.Y. (2006). Polymer electrolyte membrane based on 2-acrylamido-2-methyl propanesulfonic acid fabricated by embedded polymerization. J. Power Sources.

[20] Ramaiyan K., Bhalchandra A.K., Vijayamohanan K.P. (2008). Polymer Electrolyte Fuel Cells Using Nafion-Based Composite Membranes with Functionalized Carbon Nanotubes13. Angew. Chem. Int. Ed..

[21] Chanda M., Roy S.K. (2007). Plastic Technology Handbook.

[22] Pivovar B.S., Wang Y., Cussler E.L. (1999). Pervaporation membranes in direct methanol fuel cells. J. Membr. Sci..

[23] Pei H., Hong L., Lee J.Y. (2006). Embedded polymerization driven asymmetric PEM for direct methanol fuel cells. J. Membr. Sci..

[24] Norsten T.B., Guiver M.D., Murphy J., Astill T., Navessin T., Holdcroft S., Frankamp B.L., Rotello V.M., Ding J. (2006). Highly Fluorinated Comb-Shaped Copolymers as Proton Exchange Membranes (PEMs): Improving PEM Properties Through Rational Design. Adv. Funct. Mater..

